# Automatic Design of Synthetic Gene Circuits through Mixed Integer Non-linear Programming

**DOI:** 10.1371/journal.pone.0035529

**Published:** 2012-04-20

**Authors:** Linh Huynh, John Kececioglu, Matthias Köppe, Ilias Tagkopoulos

**Affiliations:** 1 Department of Computer Science, University of California Davis, Davis, United States of America; 2 Genome Center, University of California Davis, Davis, California, United States of America; 3 Department of Computer Science, University of Arizona, Tucson, Arizona, United States of America; 4 Department of Mathematics, University of California Davis, Davis, California, United States of America; Tata Institute of Fundamental Research, India

## Abstract

Automatic design of synthetic gene circuits poses a significant challenge to synthetic biology, primarily due to the complexity of biological systems, and the lack of rigorous optimization methods that can cope with the combinatorial explosion as the number of biological parts increases. Current optimization methods for synthetic gene design rely on heuristic algorithms that are usually not deterministic, deliver sub-optimal solutions, and provide no guaranties on convergence or error bounds. Here, we introduce an optimization framework for the problem of part selection in synthetic gene circuits that is based on mixed integer non-linear programming (MINLP), which is a deterministic method that finds the globally optimal solution and guarantees convergence in finite time. Given a synthetic gene circuit, a library of characterized parts, and user-defined constraints, our method can find the optimal selection of parts that satisfy the constraints and best approximates the objective function given by the user. We evaluated the proposed method in the design of three synthetic circuits (a toggle switch, a transcriptional cascade, and a band detector), with both experimentally constructed and synthetic promoter libraries. Scalability and robustness analysis shows that the proposed framework scales well with the library size and the solution space. The work described here is a step towards a unifying, realistic framework for the automated design of biological circuits.

## Introduction

Synthetic biology is a nascent field with transformative potential to a variety of disciplines, ranging from development of therapeutics [1] to biofuel production [2]. Although automation is one of the conceptual pillars of synthetic biology, designs still rely on a trial-and-error and tinkering approaches. When it comes to automated biological circuit design, computer-aided design (CAD) tools have still low penetrance to biological circuit design despite notable developments in the field. Recent advances include efforts to adapt electrical engineering concepts, such as Boolean optimization and Carnaugh maps, to biological circuit design of digital functions [3], and approaches that build formal high-level languages to translate from user-defined specifications to genetic circuits that adhere to digital logic [4], [5], [6].

In the realm of analog synthetic gene design, heuristic methods such as evolutionary algorithms [7], [8] and simulated annealing [9] were employed. Relevant approaches include the exploration of the functionality space of a given library [10], library-agnostic robustness analysis to determine what mutation sites for achieving the desired functionality [11]. Notably, a deterministic optimization framework was proposed by Dasika and Manaras [12] to find synthetic constructs by using an outer approximation procedure. Despite its novelty, the capabilities of that method are limited, as it targets only steady-state problems and it cannot guarantee optimality in non-convex problems, which usually is the case in biological systems.

**Figure 1:**
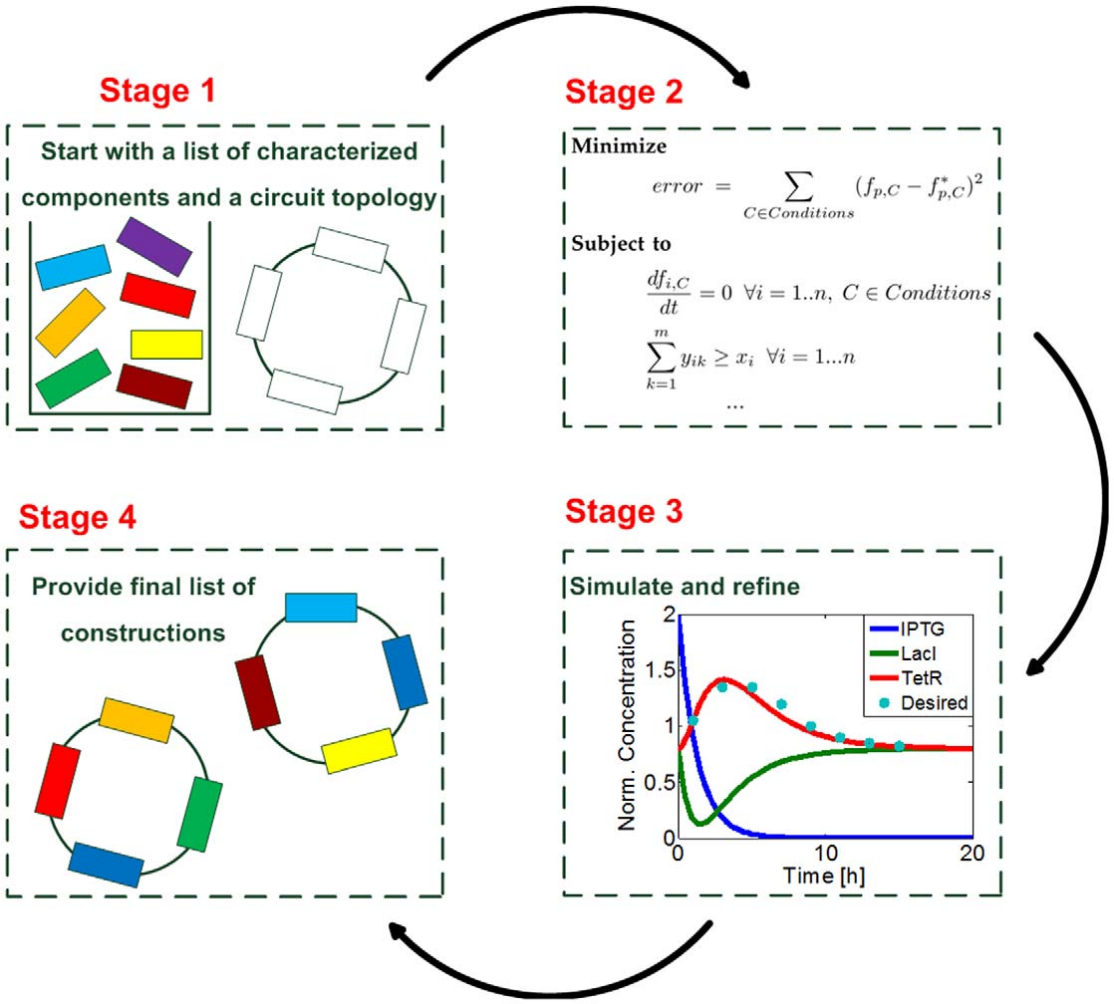
System overview of the proposed optimization framework. The software requires access to a library of characterized parts (such as a subset of the parts available in Parts Registry) that will be used as fundamental blocks in the synthetic circuit. The user will have to supply a specific design (static connectivity), together with a set of constraints and a specific objective function to be optimized. The software will translate this system to a set of linear constraints that it will subsequently solve. The result of the optimization framework will be the set of parts that have to be used, and at what position. The system will have the ability to simulate the proposed design, and provide candidate synthetic circuits for experimental construction in the laboratory.

In this paper, we focus on the problem of optimal part selection: given a library of biological parts, an objective function (e.g. a desired temporal protein profile or a dose-dependent protein expression profile), user-defined constraints (e.g. the maximum number of coding regions per promoter), and an existing abstract circuit topology, we try to find the optimal set of parts from the library so that the final circuit best approximates the objective function, given the constraints. An overview of the proposed optimization framework is illustrated in figure 1.

## Methods

### Nonlinear Model

We first describe a non-linear model that incorporates regulation, degradation, transcription and translation, and allows multiple gene copies with distinct regulation to be present in the genetic circuit. Let 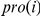 be the set of all promoters that are upstream of the one or more copies of gene 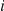. The various promoters may include transcription factor binding sites (TFBS) that will be part of the 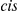-regulatory region of a gene. For each promoter 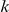 in 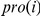 the (possibly empty) sets 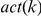 and 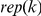 contain all activator and repressor proteins that are present in promoter 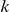, respectively. Using Hill equations (see [13], [14], [15] and [16]), the concentration of protein 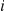 can be modeled as an ordinary differential equation (ODE) as follows:
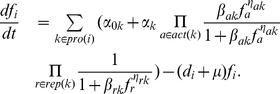
(1)where 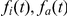 and 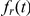 are the concentration at time point 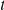 of proteins 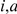 and 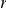, respectively. For each promoter 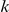 in 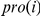, 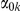 and 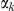 are its basal production and protein synthesis coefficient, 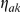 and 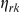 are the cooperativity coefficients for activator 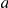 and repressor 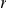, 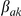 and 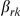 are the binding affinities of activator 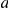 and repressor 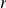. The degradation of protein 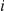 is captured by parameter 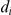. The growth rate is represented with 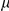, and it is considered to be zero in stationary phase.

In many cases, gene expression is controlled by exogenously applied chemicals that induce gene expression through molecular binding. We can incorporate the effect of inducers by explicitly modeling the total amount of any protein 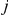 in the cell as the sum of the free (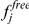) and inducer-bound protein (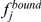), which results in the following Hill equation model:
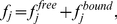
(2)

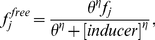
(3)

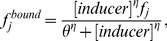
(4)where 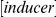 is the inducer concentration, 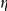 is the Hill coefficient (cooperativity factor) and 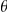 is the dissociation constant. Note that equations 2 to 4 apply for both activators and repressors, and in cases where binding of the inducer renders the transcription factor either active or inactive. For example, when inducer binding to the transcriptor factor activates transcription (as it is the case with AraC and arabinose), then the activator concentration 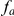 in the RHS of equation 1 is given by 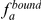 from equation 4.

Equation 1 provides a non-linear representation of protein concentration, which can be combined with binary variables that correspond to the presence/absence of a specific promoter in the synthetic circuit to formulate an optimization design problem. We introduce the following equation to express the concentration of protein 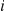 as a function of the available promoters and proteins:
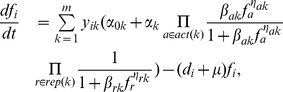
(5)where 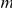 is the number of all promoters and binary variables 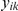 represent the presence or absence of promoter 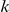 upstream of gene 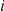:
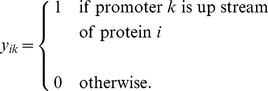
(6)


### Linear Model

The non-linear model formulation works well when the objective function is to approximate a *steady-state* expression profile since setting the derivative in equation 1 to zero results in a polynomial equation. However, approximating temporal profiles through a system of non-linear differential equations that incorporate integer variables (e.g., 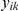) lead to a mixed integer dynamic optimization (MIDO) problem, which cannot be solved efficiently [17].

To overcome this challenge, we introduce a linearization of the non-linear model that was given in (5) by using a linear approximation around a steady-state point [18]. Approximating the model through taking the first terms of its Taylor expansion, and then incorporating the binary selection variables 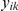 that we introduced in eq. 6 yields:
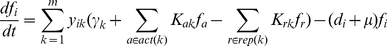
(7)where 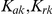 are coefficients of first order terms in the Taylor expansion over variables 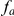 and 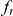 in equation 5, and 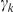 is the residual constant. Assuming 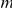 promoters and 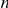 proteins total in the library, we can reformulate the above expression (eq. 7) by introducing the parameter 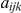 as the regulatory effect of protein 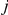 to the expression of gene 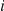 when 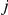 is bound on the upstream promoter 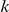 of 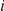 (i.e., 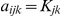 if 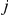 is an activator of 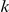, 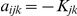 if 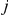 is a repressor of 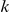, and 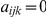 if 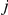 is neither an activator nor a repressor of 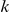):
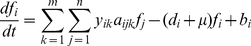
(8)where
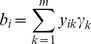
(9)


Equation 8 described the protein production rate for any protein in a closed protein set 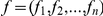. To solve this linear system, we re-write it in its matrix form, as follows:
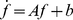
(10)where the elements of the 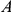 matrix are defined as:
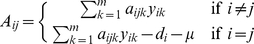
(11)and 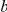 is given by
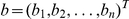
Assuming that matrix 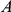 is invertible, the analytical solution of this equation is as follows [19]:
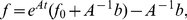
(12)where 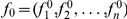 are the initial concentrations of the proteins 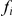 in the closed set 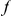. In cases where matrix 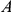 can be diagonalized, then the term 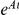 in equation 12 is given by:
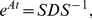
(13)where 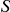 is the matrix which columns are the eigenvectors of 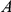, each corresponding to a distinct eigenvalue 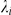, and 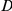 is the diagonal matrix, where the diagonal elements are equal to 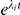. The diagonalization of matrix 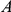 can be achieved in many special cases (e.g., when the characteristic polynomial is simple, the eigenvalues can be explicitly calculated). For the scenarios when this is not feasible, we can approximate 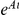 by taking its Taylor expansion, although this can be computationally intensive if high accuracy is needed [20]:
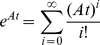
(14)


The linearization of the non-linear model, as described in this section, provides an efficient method to approximate non-linear temporal dynamics. However, it may perform poorly when the dynamics of the system to optimize are highly non-linear (oscillatory behavior, bi-stability, etc.). In such cases, we can divide the desired temporal profile into multiple domains/intervals, under which the linear system can better approximate the non-linear dynamics. By solving the optimization problem over multiple intervals, the algorithm is able to compute candidate solutions with higher accuracy, at the cost of higher time and space complexity. To ensure continuity during optimization of calculated protein concentration in successive intervals, the initial concentration 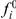 of protein 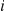 at any interval can be set to be equal to the final protein concentration in the preceding interval. In this paper, we use this setup for the temporal profile optimization of the toggle switch design.

### Steady state optimization

In the case of steady-state optimization, our task is to design a genetic circuit in which one or more proteins operate at a specific concentration values, that may be given as a function of an exogenous parameter (e.g., inducer concentration). In the context of MINLP, the formulation of the problem is as follows:

**Minimize**

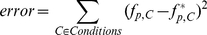
(15)


**Subject to**

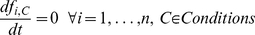
(16)

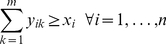
(17)

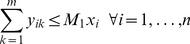
(18)

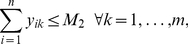
(19)where 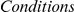 is the set of the desired input/output value pairs that are given, 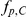 and 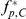 are the estimated and the desired steady state concentration of a protein 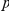 in condition 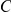, respectively. The binary variable 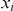 captures the presence or absence of gene 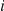 in the circuit. 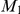 and 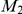 are the maximum number of promoters at the upstream of each gene copy, and the maximum number of genes downstream of each promoter, respectively. The first constraint (eq. 16) represents the steady state condition by setting the LHS of equation 5 to be zero for all conditions (e.g., different inducer concentrations). The next two constraints ensure that there will be at least one promoter for each gene (eq. 17), but none for a gene that is not a part of the genetic circuit (eq. 18). The last constraint is optionally given by the user and it is used to limit the maximum number of genes in an operon (eq. 19).

### Temporal profile optimization

In the case of finding the components of the genetic circuit that best approximates a temporal profile, the MINLP problem is formulated as follows:

**Minimize**

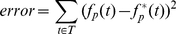
(20)


**Subject to**

(7–12)

(13) or (14)

(17–19)

where 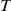 is the set of time points, 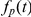 and 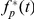 are the estimated and the desired concentration of a protein 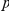 at a time point 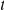.

## Results

Both the steady-state and the temporal profile optimization problems can be solved by using global mixed-integer non-linear solvers that rely on linearization, convexification, and application of branch and bound methods. Here, we used the COUENNE 0.4.0 open-source platform [21], which we extended in scope to handle the problems that we focus on: we decoupled termination conditions for the primal-dual gap, and modified the updating condition in the bound tightening procedure by introducing threshold parameters. To evaluate the capacity of our optimization framework to yield synthetic circuits with the desired characteristics, we assessed its performance in three synthetic circuits that have been constructed experimentally: a band detector system [22]–[23], a transcriptional cascade [24], and a toggle switch [25]. For all designs, we used parameter values that were previously reported in literature (Table 1), and the initial protein concentrations where assumed to be zero. Regarding the experimentally characterized part mutant libraries that we used [26]–[28], all promoter mutants differ in their basal level of production 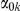 and the protein synthesis coefficient 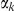. In order to evaluate the scalability of the framework, we constructed synthetic libraries that consisted of synthetic promoter parts with parameter values within the experimentally measured range, with a sampling distribution that varied from uniform to gamma (more details below).

**Table 1:** Parameter values.

Description	Notation	Min	Max	Value	Units	References
Duality gap threshold (COUENNE)	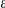	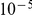	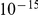			
Protein synthesis coefficient						
Constitutive promoters	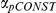	0.1	25.5		au/h	[27] [30]
LAC promoters	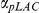	0.3	7.1		au/h	[26] [30] [23]
TET promoters	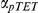	0.3	9.2		au/h	[26] [30]
BAD promoters	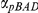	2.8	3.4		au/h	[28]
Basal production						
LAC promoters	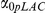	0.003	0.2		au/h	[26] [30]
TET promoters	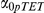	0.003	0.03		au/h	[26]
BAD promoters	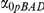	0.002	0.005		au/h	[28]
Binding affinity						
LacI & LAC promoter	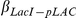			1296	au 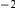	[30]
CRP & LAC promoter	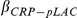			27	au 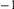	[31]
TetR & TET promoter	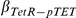			720	au 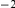	[30]
AraC & BAD promoter	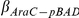			10800	au 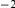	[28]
Cooperativity coefficient						
LacI	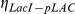			2		[23]
CRP	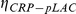			1		[31]
TetR	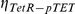			2		[32]
AraC	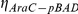			2		[12]
IPTG	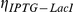			2		[25]
aTc	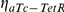			2		[25]
L-arabinose	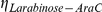			2		[28]
Degradation rate						
LacI	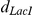	0.9	8.3	0.9	1/h	[33] [23] [30]
TetR	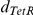	1.5	8.3	1.5	1/h	[33] [30]
AraC	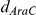			0.69	1/h	[34]
CRP	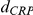			0.7	1/h	[35]
GFP	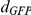	0.7	4.2	1.04	1/h	[36] [23]
yEFP	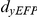			0.9	1/h	[30]
Dissociation constant						
IPTG	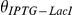			30	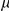 M	[25]
aTc	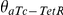			26.3	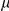 M	[8]
L-arabinose	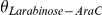			2.8	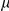 M	[28]

Parameter values that were used for the evaluation, and literature reference where they are reported. In the case where values are normalized, arbitrary units (“au") are used.

### Band Detector Design

**Figure 2:**
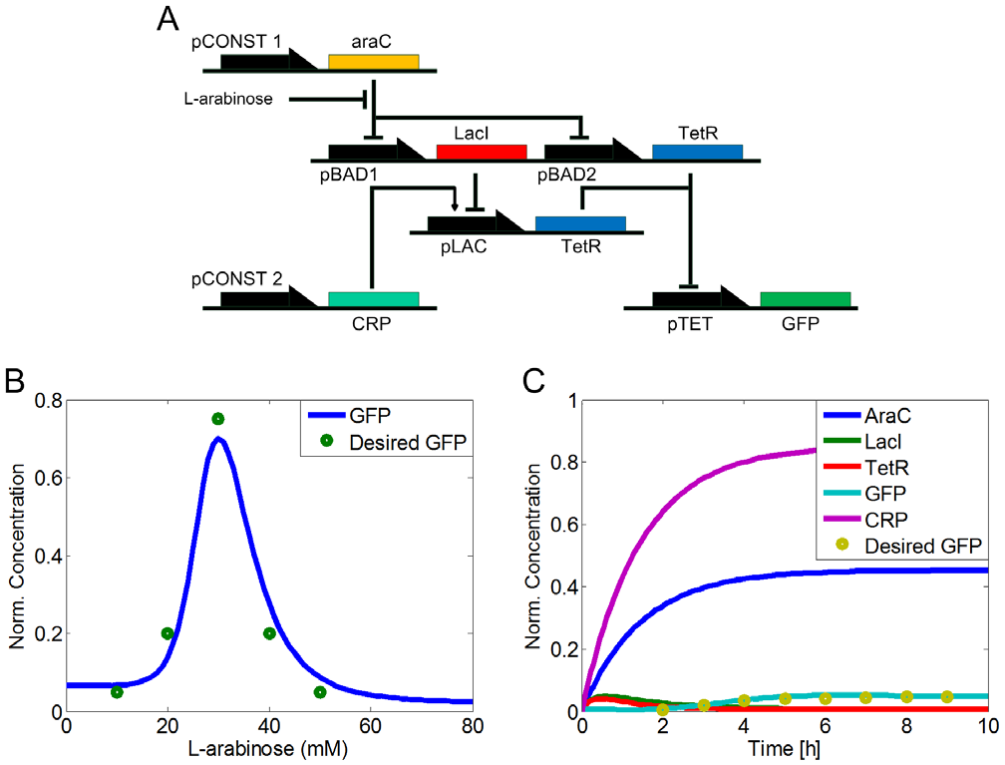
Band-pass filter design. A) The system will only express the reporter when the concentration of the input signal (L-arabinose) is in a specific range. In this design, pCONST1 and pCONST2 are constitutive promoters, while pBAD1, pBAD2 and pLAC are the promoters where AraC and LacI bind, respectively. There are two coding regions of TetR which are put on the downstream of promoters pBAD2 and pLAC. In the absence of L-arabinose, AraC activates TetR production by de-repressing the pLAC promoter. In high L-arabinose concentrations, TetR is again produced through the de-repression of the pBAD2 promoter. In significant, but not high inducer concentrations, however, none of the pathways are active enough, which in turn results in lower TetR levels and subsequent expression of the reporter GFP output. B) Reporter protein concentration (output) versus L-arabinose levels (input). The output of the synthetic circuit becomes high only at moderate values of L-arabinose. Green circles denote desired values (fluorescence measurements) that act as input to our optimization platform. C) Temporal expression profile of the band-pass filter. Temporal profile of the resulting optimal synthetic gene circuit, for a L-arabinose level of 30 mM. The GFP concentration of the optimization-derived circuit (cyan solid line) matches well the desired input values (yellow solid dots).

We used the MINLP optimization framework to find the optimal combination of promoters for a six-promoter bandpass design that acts as a filter: the output is high only when the input is within a specific range or “band". The first bandpass synthetic design was used to detect acyl-HSL signal in a population of bacteria [22]–[23]. In [12], a simpler design to detect L-arabinose within a bacterium was introduced (Fig. 2A). The mode of operation for this circuit is the following: The output, a GFP reporter protein is only high when TetR protein is not present. There are two pathways that produce TetR, one directly through activation of the pBAD promoter, and another through the LacI de-repression of the Lac promoter. When the concentration of L-arabinose is high, L-arabinose will bind to AraC and prevent it binding to pBAD and repress its expression. This results in TetR expression through the pBAD-TetR pathway. At the same time, LacI will also be expressed and it will repress the pLAC-TetR production pathway. Similarly, the opposite is observed at low concentrations of L-arabinose, where the pLAC-TetR pathway is activated and the pBAD-TetR pathway repressed. Therefore, TetR will be expressed for both cases: low or high concentration of L-arabinose. However, because of the difference in the regulation of pBAD and pLAC, there will be a value interval of L-arabinose that the expression level of TetR is low, and the GFP reporter protein is expressed (Figure 2A).

A dataset with experimentally characterized promoters [27]–[29] of various strengths and types (constitutive, pBAD, LacI, TetR) was used as the library of parts available. Model parameters were set on literature reported values for *E. coli* and are summarized in Table 1. In the original band-detector circuit, the objective function is a steady-state I/O characteristic between the input (inducer L-arabinose) and the output (reporter GFP), with no specification on the transient characteristics of the system. The MINLP optimization method was able to find the optimal combination of parts for the steady-state case within minutes (Figure 2B). Similar results were obtained for temporal profile optimization at a L-arabinose concentration of 30 mM by using the linear model described above (Fig. 2C). The optimality of the solution was verified by running exhaustive search.

### Transcriptional cascade design

**Figure 3:**
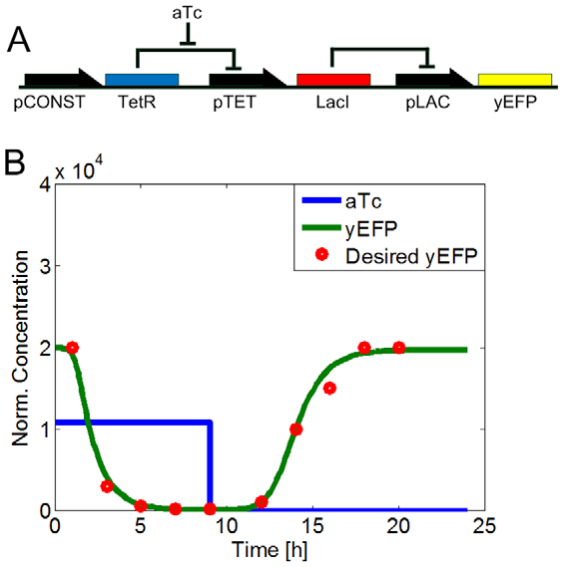
Transcriptional cascade design. A) The system is controlled by the inducer aTc which can bind to TetR and reduce the concentration of free TetR molecules. This concentration change will be propagated through the cascade to the change of the reporter yEFP. B) Temporal profile of a cascade design: The desired output (yEFP, red dots) and the actual output (green line) of the optimal synthetic gene circuit are showed. The temporal profile was split into two phases, based on changes in the inducer concentration. In the first phase (0 h–9 h), 2.16 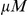 aTc (blue line) is added and in the second phase (9 h–24 h) aTc is washed out (setting as the experiment in [24]).

Next, we used the MINLP optimization framework to identify optimal part combinations for the temporal profile of a transcriptional cascade design that was proposed in [24]. According to this design, TetR is under a constitutive promoter and it represses LacI expression, which in turns represses yEFP. (Fig. 3A). At normal conditions, TetR will be created and bind to the pTET promoter to prohibit LacI production and thus the expression level of yEFP is high. When the inducer aTc is added, this inducer will bind to TetR proteins and prevent them binding to the pTET promoter and thus the production of LacI from this promoter will be maximized and the expression level of yEFP is low. If the inducer aTc is washed away, the system returns to the initial condition and the expression level of yEFP is high. Previously, we used the promoter library from [26] and [27] as inputs to our optimization framework. The time course is divided into 2 phases, based on the presence of the inducer aTc. The characteristic function of the resulting optimal design is showed in figure 3B.

### Toggle switch design

**Figure 4:**
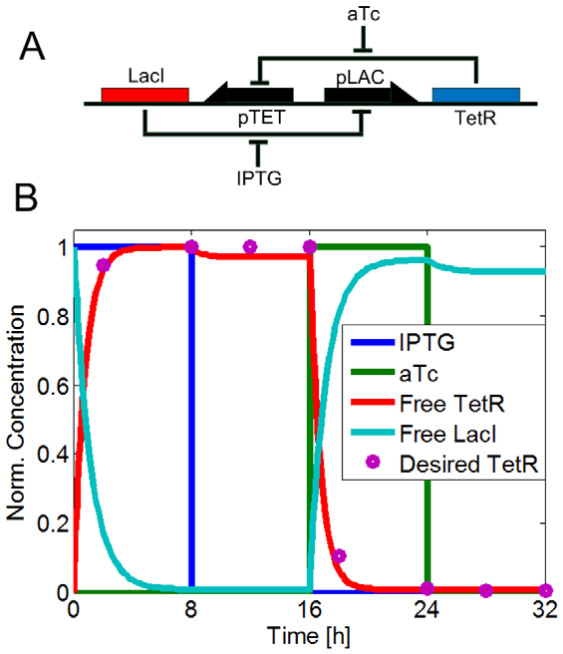
Toggle switch design, where two genes (LacI and TetR) negatively regulate each other. A) The system is externally controlled through the addition of two inducers, IPTG and aTc, which bind to the repressors and decrease their regulatory potential. B) Expression profile of the resulting synthetic circuit: The desired profile (input, depicted with purple dots) and actual profile (red line) for the TetR protein is shown. The temporal profile was split into four phases, based on changes in the inducer concentrations. Phase 1: IPTG high, aTc low; Phase 2: IPTG low, aTc low; Phase 3: IPTG low, aTc high; Phase 4: IPTG low, aTc low.

Toggle switches, also known as flip-flops, are fundamental memory blocks that have two stable attractor points, where one of the outputs is high and the other is low. As a test case we used the toggle switch design from [25]. As shown in figure 4A, the design has two genes, LacI and TetR, that negatively regulate each other. This is possible through the addition of a LacI promoter in front of the TetR gene (denoted as pLAC), and a TetR promoter in front of the LacI gene (denoted as pTET). In addition, the system can be controlled by the chemical inducers IPTG and aTc that can shut down the repressing effect of LacI and TetR, respectively.

The toggle switch has two-attractor dynamics, as shown in Figure 4B: The system initially is in one of the two steady states, with either LacI or TetR overexpressed. When the system is induced with IPTG, the inducer binds to LacI and it suppresses its regulatory activity upon the TetR production (phase 1). This leads to the overexpression of TetR gene (which is now de-repressed, in the absence of LacI), that in turn shuts down the LacI production, by binding to its promoter and acting as a repressor. So, even when IPTG is washed away from the system (phase 2), LacI remains repressed. Subsequent addition of the aTc inducer results to its binding to TetR protein, changing it conformation and thus, de-repressing the LacI protein, which now is free to start repressing the TetR expression (phase 3). Once this reaches a steady state, it remains at that state, even at the removal of the inducer aTc (phase 4). A mutant library for the Tet and Lac promoters was used as before [26]. The objective function was set to be the transient dynamics of a toggle switch with respect to the TetR protein, as shown in figure 4B. As discussed in the methods section, in order to better approximate the temporal profile of this circuit, its profile was split in four phases as dictated by the various inducer concentrations.

### Method evaluation: approximation error, running time and scalability analysis

#### Approximation error and running time

Table 2 summarizes the approximation error and running time of exhaustive search (ES), a genetic algorithm heuristic (GA) and the proposed mixed-integer non-linear programming (MINLP) approach on all three design problems. To increase the likelihood that the GA will find the globally optimal solution, we performed a number of initial point randomizations and kept the heuristic running time within the same order of magnitude as the MINLP method. For the latter, we allowed the solution to be near-optimal with a duality gap (i.e., a guaranteed upper bound on the approximation error) of less than 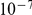. As it is shown in Table 2, both the GA and MINLP method were able to find optimal or near-optimal solutions much faster than exhaustive search. In addition, MINLP outperforms the GA heuristic in all steady-state cases, and it performs on par or better in all temporal optimization cases. However, we stress again that the major advantage of MINLP is that it can *guarantee* the optimality of the solution, or its maximum deviation from such optimal point, something that heuristics are unable to provide.

**Table 2:** Comparison of the approximation error and running time.

Design	Library	Running	Optimal	Running	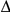 Error	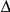 Error	Running	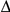 Error
	Size	Time	Error	Time	Min	Max	Time	Final
		(ES)	(ES)	(GA)	(GA)	(GA)	(MINLP)	(MINLP)
Band detector								
Steady state	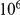	1.2 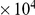	1.3 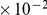	1.2 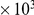	0	1.9 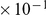	1.5 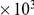	0
Temporal	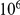	5.0 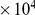	3.3 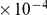	2.0 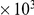	1.0 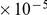	2.3 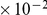	2.0 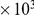	1.6 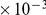
Cascade								
Steady state	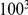	7.3 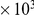	3.8 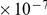	8.2 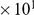	5.6 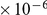	1.4 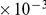	7.1 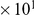	4.0 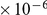
Temporal	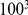	8.7 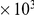	1.5 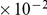	1.2 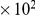	0	1.5 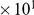	1.8 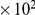	4.4 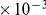
Toggle Switch								
Steady state	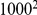	2.5 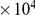	8.2 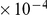	1.0 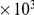	3.2 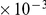	4.7 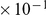	9.9 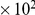	0
Temporal	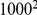	3.2 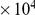	1.8 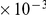	1.3 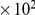	0	5.0 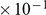	7.7 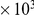	1.7 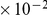

A comparison of the running time (seconds) and the optimality of the exhaustive search (ES) method, the genetic algorithm (GA) heuristic and the proposed mixed-integer non-linear programming approach (MINLP). “Optimal error" refers to the squared difference between the desired protein value and the optimal circuit value, when the later was found through exhaustive search. “
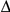
Error" refers to the difference between the optimal error and the heuristic or MINLP error. Since the genetic algorithm solution depends on the initial conditions, “
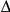
Error Min" and “
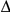
Error Max" are given.

#### Scalability and sensitivity analysis

**Figure 5:**
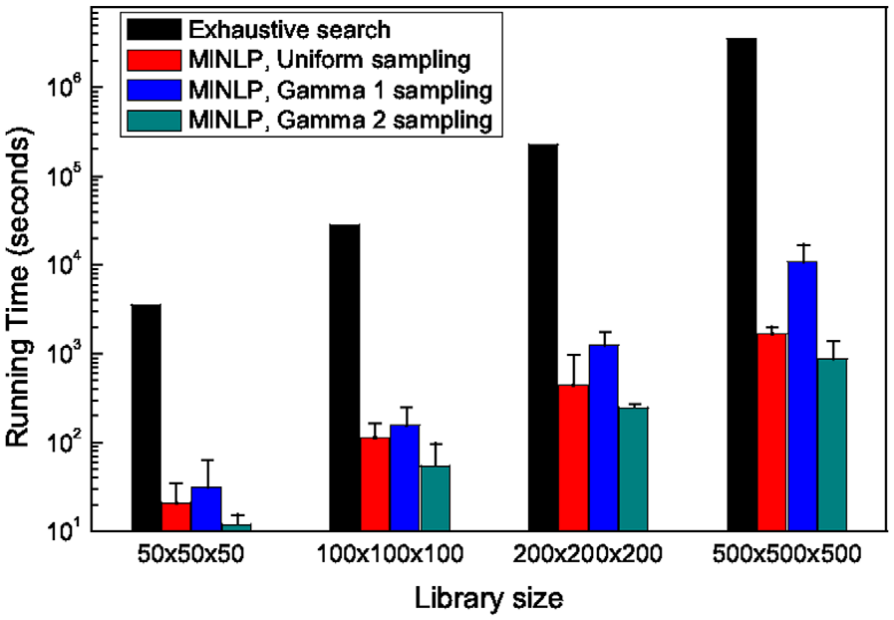
Scalability and sensitivity of the running time of the MINLP approach on different inputs. A comparison of the running time (seconds) of the MINLP approach on different input sizes and different data input distribution for the steady state cascade design problem (5 synthetic datasets per case). Parameter values are generated by sampling within the parameter range that has been experimentally measured [26]–[27]. Samples are distributed either uniformly (uniform sampling), following a gamma distribution with the mean set on the optimal set parameter values (Gamma 1 sampling), or away from that mean (Gamma 2 sampling). The running time of exhaustive search method (ES) is estimated for the last library size.

In order to measure the scalability of MINLP approach, we have evaluated it on the cascade design with different input library sizes as in figure 5. As shown in the table, the ratio between ES to MINLP running time increases considerably as the library size scales up. In addition, since the MINLP problem is solved by a branch-and-bound algorithm, the distribution of part values may affect the running time of the algorithm. To check the sensitivity of our MINLP framework on the distribution of parts in the library, we generated three synthetic libraries. In the first, the part values were uniformly distributed within the parameter range. In the second, the parts where gamma distributed with a mean near the parameters of the optimal solution (identified by the previous experiment). Similarly, in the third library, the part values are gamma distributed with a mean that is far from the optimal part parameters. As it is evident from figure 5, high density of parts in the region of the optimal solution leads to inferior performance (about an order of magnitude for all library sizes), since the existence of many near-optimal solutions render the branch-and-bound task difficult. Similarly, the inverse is observed when the part values are not close to the optimal solution. Nevertheless, the performance of the algorithm was in all cases orders of magnitude better than the exhaustive case.

## Discussion

In this paper we introduced a global mixed-integer non-linear programming framework for the automatic construction of synthetic gene circuits with either steady-state or temporal objectives. Profiling, scalability and sensitivity analysis on three synthetic circuits that have been experimentally constructed in the past, show that the method compares favorably to both exhaustive search and heuristic methods. In addition, in contrast to all other techniques so far, the method presented is able to provide guarantees on the global optimality of the solution.

There are several extensions of this work that warrant further investigation. First, we will systematically investigate how the circuit topology affects the performance of this and other methods. Although our results were similar for all three topologies that we analyzed, we expect that the topological characteristics of the synthetic circuits (e.g., the number of feedback loops present) together with the parameter distribution of the parts library will play a significant role on the performance of any automatic circuit construction method. In addition, we can extend the current framework to include in the optimal set of other part types (operator sites, ribosomal binding sites, gene mutants, etc.) during the optimization procedure. Although we are currently lacking well-characterized libraries of such components, recent initiatives (such as the Biofab project) will increase the availability of such components. One formidable technical challenge is to come up with an automatic way to determine the threshold values that are related to the optimization method and tools used. For example, COUENNE uses an error threshold for bound tightening that we found to have significant effect on the number of infeasible cases that the tool reports. By adjusting this threshold we were able to decrease the number of infeasible cases to zero, at the cost of computational time. Currently there is no way to estimate the threshold value, and an adaptive iterative method may produce interesting results. Finally, the proposed framework can be extended towards *ab initio* synthetic circuit design where the circuit topology is not known. The method presented here, provides a stepping stone towards building highly efficient, pragmatic tools for synthetic circuit design.
